# Missing the forest because of the trees: slower alternations during binocular rivalry are associated with lower levels of visual detail during ongoing thought

**DOI:** 10.1093/nc/niaa020

**Published:** 2020-10-05

**Authors:** Nerissa Siu Ping Ho, Daniel Baker, Theodoros Karapanagiotidis, Paul Seli, Hao Ting Wang, Robert Leech, Boris Bernhardt, Daniel Margulies, Elizabeth Jefferies, Jonathan Smallwood

**Affiliations:** n1 Department of Psychology, University of York, York YO10 5DD, UK; n2 School of Psychology, University of Plymouth, Plymouth, UK; n3 Department of Psychology, Duke University, Durham, NC, USA; n4 Sackler Centre for Consciousness Science, University of Sussex, Brighton, UK; n5 Centre for Neuroimaging Science, Kings College London, London, UK; n6 Montreal Neurological Institute and Hospital, McGill University, Montreal, Canada; n7 Centre National de la Recherche Scientifique (CNRS) UMR 7225, Institut du Cerveau et de la Moelle epiniere, Paris, France; n8 Department of Psychology, Queen's University, Kingston, Ontario, Canada

**Keywords:** ongoing thought, binocular rivalry, detail, diffusion tensor imaging, experience sampling, fractional anisotropy

## Abstract

Conscious awareness of the world fluctuates, either through variation in how vividly we perceive the environment, or when our attentional focus shifts away from information in the external environment towards information that we generate via imagination. Our study combined individual differences in experience sampling, psychophysical reports of perception and neuroimaging descriptions of structural connectivity to better understand these changes in conscious awareness. In particular, we examined (i) whether aspects of ongoing thought—indexed via multi-dimensional experience sampling during a sustained attention task—are associated with the white matter fibre organization of the cortex as reflected by their relative degree of anisotropic diffusion and (ii) whether these neurocognitive descriptions of ongoing experience are related to a more constrained measure of visual consciousness through analysis of bistable perception during binocular rivalry. Individuals with greater fractional anisotropy in right hemisphere white matter regions involving the inferior fronto-occipital fasciculus, the superior longitudinal fasciculus and the cortico-spinal tract, described their ongoing thoughts as lacking external details. Subsequent analysis indicated that the combination of low fractional anisotropy in these right hemisphere regions, with reports of thoughts with high levels of external details, was associated with the shortest periods of dominance during binocular rivalry. Since variation in binocular rivalry reflects differences between bottom-up and top-down influences on vision, our study suggests that reports of ongoing thoughts with vivid external details may occur when conscious precedence is given to bottom-up representation of perceptual information.

**Highlights**

- Greater fractional anisotropy (FA) in right hemisphere is linked to less detailed task-related thought.
- Individuals with shortest binocular rivalry dominance have low FA and more detailed ongoing thoughts.
- Detailed task-related thoughts may occur when precedence is given to bottom-up perceptual information.

## Introduction

Conscious experience varies from moment to moment, and studies using experience sampling suggest that these dissociations can take multiple forms. Sometimes our thoughts switch away from an external task to become focused on personal experiences rather than events in the external environment, or any task being performed (Smallwood and Schooler 2006, 2015; Seli *et al.* 2018). Research suggests states of off-task thought are linked to systems important for attentional focus (Hasenkamp *et al.* 2012; Turnbull *et al.* 2019a,b). In particular, it has been recently demonstrated that the ventral attention network is associated with an individual’s ability to regulate the focus of attention during sustained attention in a manner that accounts for the demands imposed by the external environment (Turnbull *et al*., 2019a, b), a phenomenon known as context regulation (Smallwood and Andrews-Hanna 2013).Using experience sampling in conjunction with the simultaneous recording of neural activity using functional Magnetic Resonance Imaging (fMRI), Turnbull *et al.* (2019a) found that activity in the dorsal prefrontal cortex (BA 9/46) was associated with both being on-task during a working memory task, and also associated with being off-task in a situation when the task demands were lower. In contrast, the same study highlighted that regions of parietal cortex, in the dorsal attention network, was linked to reports of on-task experience across both conditions. We confirmed the dissociations between these two large-scale networks in separate cohort study which found that connectivity of the ventral attention network with sensorimotor cortex was linked to the alignment of experience with task goals (i.e. more on-task experience when task demands are higher, and more off-task thought when demands are lower). In contrast, connectivity of the dorsal attention network with regions of lateral occipital cortex was linked to the on-task state regardless of task context (Turnbull *et al.* 2019b). This dissociation between different attentional systems in terms of the prioritization of different types of thought, and the representation of different forms of informational content are broadly consistent with theoretical perspectives on the neural basis of different features of ongoing thought (Smallwood and Schooler 2015; Christoff *et al.* 2016).

Experiences, however, can also fluctuate in the level of detail with which events in the external environment are processed. Recent studies using experience sampling suggest that detailed experiences during sustained attention can depend upon the functioning of the default mode network (DMN). In particular, we found that patterns of detailed thought are more pronounced during working memory tasks and representational similarity analysis demonstrated that, under these conditions, neural signals within regions of the DMN encode this feature of experience (Sormaz *et al.* 2018). Moreover, individuals who have more detailed task experiences in the laboratory in general show stronger coupling between the DMN and regions of visual cortex (Turnbull *et al.* 2019b), recruit the posterior cingulate more during periods of self-reference (Murphy *et al.* 2019) and show greater cortical thickness in the para-hippocampus (Ho *et al.* 2019). Although a link between the DMN and externally focused experience is surprising given the widely held assumption that the DMN was limited to internally focused, task unrelated experiences (e.g. Fox *et al.* 2005), this proposed link is nonetheless consistent with more recent findings. For example, studies indicate that people can perform tasks with high levels of efficiency when they are ‘in the zone’ (Esterman *et al.* 2013; Kucyi *et al.* 2016) or ‘on autopilot’ (Vatansever *et al.* 2017a). Thus, understanding the neural mechanisms underlying different patterns of experience is not only important for contemporary accounts of ongoing conscious thought (Smallwood and Schooler 2015) but may also be important for appropriately characterizing the function of different large-scale neural networks.

The current study aimed to elucidate the role that top-down visual processes play in different types of dissociation between ongoing experiences and environmental events. Traditionally, research into conscious experiences has emphasized that it is possible to understand the relationship between subjective awareness and the immediate sensory context using situations of bistable perception (such as the Necker Cube, or the phenomenon of binocular rivalry) because, in such contexts, awareness can change without a concomitant change in sensory input (Crick 1996). Situations of bistable perception provide relatively unambiguous indices of the top-down influence on vision because they discard low-level processes that contribute to the process of perception (e.g. sensory transduction). In this context, the dominance of one image during bistable perception is assumed to reflect the influence of top-down processes on vision. Consistent with the assumption that bistable perception depends on the balance between top-down and bottom-up influences on vision, neuroimaging studies suggest that rivalry depends on both processes taking place in visual regions (Tong *et al.* 2006) as well as higher-order brain regions (Knapen *et al.* 2011; Baker *et al.* 2015). Importantly, both default mode and attention systems are important in binocular rivalry: whereas disruptions to regions of the DMN, such as the posterior parietal lobule, tend to lengthen perceptual alterations during bistable perception (Carmel *et al.* 2010; Kanai *et al.* 2011), disruptions to nearby regions of parietal cortex, within the dorsal attention network, shorten perceptual alterations (Kanai *et al.* 2011).

Our study sought to extend our understanding of naturally occurring changes in ongoing experience by linking them to both changes in the structural organization of the cortex and to indices of the top-down influence on vision as estimated from binocular rivalry alternations. Specifically, we analysed data from a large cohort of individuals who had extensively described the contents of their ongoing experience during a laboratory task (for prior publications, see Sormaz *et al.* 2018; Wang *et al.* 2018; Ho *et al.* 2019; Turnbull *et al.* 2019a,b) and for whom we also acquired measures of binocular rivalry using a paradigm similar to that used in our prior study (see Baker *et al.* 2015). These individuals also had measures of structural connectivity provided by diffusion tensor imaging (DTI), which has highlighted neural processes linked to both binocular rivalry (Genç *et al.* 2011) and to patterns of ongoing thought in a prior study (Karapanagiotidis *et al.* 2017). In the study by Karapanagiotidis *et al.*, we found a right-lateralized region of white matter that had greater fractional anisotropy (FA) for individuals who tended to neglect the external environment to imagine events in the past or future instead of those in the here and now. The current study aimed to replicate the association between ongoing experience and the white matter architecture of the right hemisphere in a new set of participants, and explore whether this association was related to the relative balance between top-down and bottom-up influences on vision during binocular rivalry, as indexed by an individual’s reported experience during binocular rivalry.

**Figure 1.**
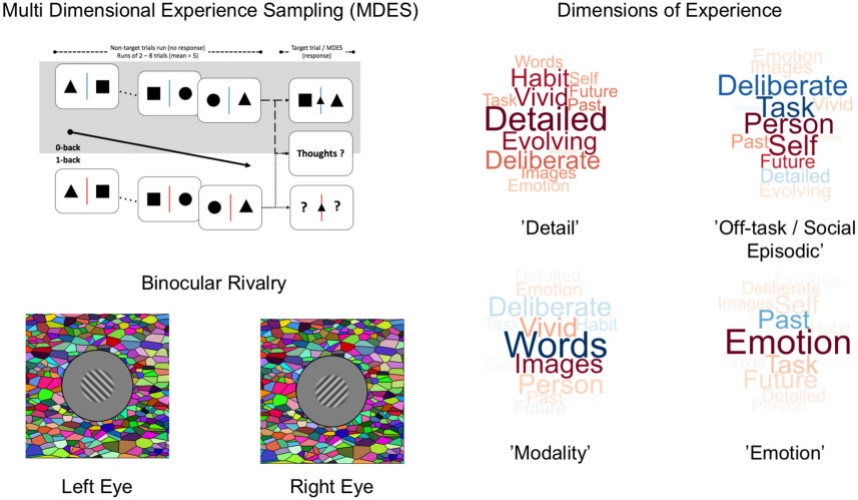
Experimental protocol. Participants participated in laboratory sessions in which we used experience sampling while they performed a simple cognitive task (top left), and in which we measured their conscious experiences through the estimation of binocular rivalry during bistable perception (bottom left). Application of Principle Component Analysis (PCA) to the Multi-Dimensional Experience Sampling (MDES) revealed four components, which are displayed in the form of word clouds on the right hand side panel. The colour and size of the words indicate the loadings of each question (font size = strength of relationship and colour = direction: warm is positive, cool is negative). The labels we used to describe these components in the paper are presented in quotations

The left hand panel in Fig. 1 describes the paradigm we used to measure ongoing experience during a sustained attention task (top left), as well as the stimuli we used in a separate session to measure binocular rivalry (bottom left). Using these data, in combination with metrics of white matter architecture provided by DTI, we set out to answer two questions: (i) Are individual differences in the pattern of one’s ongoing thoughts reflected in the structure of cortical white matter? And (ii) Are the neurocognitive descriptors for the patterns of ongoing thought related to more precise descriptions of perceptual experience as assessed by measures of binocular rivalry? It is important to note that, in our study, we did not measure ongoing experience during binocular rivalry.

## Materials and Methods

### Participants

One hundred and fifty healthy, right-handed, native English speakers, with normal or corrected-to-normal vision and no history of psychiatric or neurological illness (mean age = 20.19 and 92 were females) participated in the study. All participants had provided their written informed consent approved by the Department of Psychology and York Neuroimaging Centre (YNIC), University of York ethics committees, and were debriefed after completion of the study. Participants were either paid or given course credits for their participation.

### Procedures

Participants arrived at YNIC where we acquired brain images including T1-weighted magnetic resonance imaging (MRI), resting state MRI and DTI. On subsequent days, participants took part in a comprehensive set of behavioural assessments that captured different aspects of cognition, including both the experience sampling task and other experimental tasks (including binocular rivalry). These tasks were completed over three sessions on different days, with the order of sessions counterbalanced across participants. The task in which ongoing experience was measured always took place at the beginning of these laboratory sessions.

### Experience sampling

We measured patterns of ongoing cognition in a paradigm that manipulated memory load by using alternating blocks of 0-back (low-load) and 1-back (high-load) conditions (see top left panel of Fig. 1), with the initial block counterbalanced across individuals (see Turnbull *et al.* 2019b for a complete description of this task). Multi-dimensional Experience Sampling (MDES) was used to measure the contents of ongoing thought. On each occasion, participants reported their thoughts by responding to one of the 13 questions presented in Supplementary Table S1. Participants always rated their task focus first, and then described their thoughts at the moment before the probe on a further 12 dimensions. Participants always answered all questions and were probed on an average of 27 occasions during the task over the three sessions of the experiment. The rationale behind our approach is that different patterns of thought can be identified as regularities in covariation with how the questions are answered. These patterns can be quantified by applying statistical techniques, such as principal components analysis (PCA), to the experience sampling data. In this context, the dimensions produced by the application of PCA to MDES data acted as proxies for different thought patterns. Prior studies have shown that the patterns identified in this manner are robust to different samples of participants (Smallwood *et al.* 2016), consistent across situations (e.g. during scanning and in the behavioural laboratory, Sormaz *et al.* 2018) and show a degree of correspondence between experiences in the real world and in the laboratory (Ho *et al.* 2020).

### Binocular rivalry

We showed rivalling stimuli to participants for four trials of 120 s in duration and asked them to report their percepts using a computer mouse. The stimulus consisted of oblique gratings (1c/deg, 50% contrast, ±45 deg, 6 deg in diameter, smoothed by a raised cosine envelope) shown to opposite eyes (see bottom left panel of Fig. 1). All stimuli were presented on a gamma-corrected Iiyama VisionMaster Pro 510 cathode-ray tube (CRT) monitor with a mean luminance of 32 cd/m^2^ and were viewed through a mirror stereoscope to permit presentation of different images to the left and right eyes. The stimuli were surrounded by a dark ring and a binocular Voronoi texture to promote binocular vergence and fusion (Baker and Graf 2009). Participants held down one mouse button when they perceived a particular percept (e.g. a left-oblique grating) and the other when they perceived the alternative (e.g. a right-oblique grating). If they simultaneously perceived both percepts, or experienced a mixed percept, they held down both buttons. This allows our paradigm to reveal the duration of time in which one percept dominated the other, as well as situations when both images were perceived at the same time. We counterbalanced the orientations of the rivalling stimuli between the eyes on alternate trials.

### Diffusion tensor imaging

The DTI scan lasted 13 min. A single-shot pulsed gradient spin-echo echo-planar imaging (EPI) sequence was used with the following parameters: *b* = 1000 s/mm^2^, 45 directions, 7 T2-weighted EPI baseline scans, 59 slices, FOV = 192 × 192 mm^2^, TR = 15 s, TE = 86 ms (minimum full), voxel size = 2 × 2 × 2 mm^3^, matrix = 96 × 96. DTI data preprocessing steps involved eddy-current distortion correction and motion correction using FMRIB's Diffusion Toolbox (FDT) v3.0, part of FMRIB Software Library (FSL) (Smith *et al.* 2004). FA was calculated by fitting a tensor model at each voxel of the preprocessed DTI data and the resulting images were brain-extracted using Brain Extraction Tool (BET) (Smith 2002). Voxelwise FA maps were analysed using tract-based spatial statistics (TBSS) (Smith *et al.* 2006). After participants’ FA data were non-linearly aligned to FMRIB58_FA standard space, they were transformed to the mean space of these subjects and then affine transformed to the 1-mm MNI152 space. Next, the mean of all FA image was created and thinned to create a mean FA skeleton representing the centres of all tracts common to the group.

The skeletonized FA images were then fed into voxelwise statistics, using FSL's randomize command (a non-parametric permutation inference tool). Using a generalized linear model (GLM), the measured FA values across the skeleton were regressed with the experience sampling results, while age and gender were included as nuisance covariates. T-statistic maps for contrasts of interest were calculated with 5000 permutations (Nichols and Holmes 2002). Resulting maps were thresholded at a family-wise error (FWE) corrected *P*-value of 0.05 using threshold-free cluster enhancement (TFCE) (Smith and Nichols 2009).

Probabilistic diffusion models were also fitted using Bayesian Estimation of Diffusion Parameters Obtained using Sampling Techniques (BEDPOSTX) (Behrens *et al.* 2003), with 2 fibres modelled per voxel for 1000 iterations. Probabilistic tractography was performed using probabilistic tracking with crossing fibres (ProbTrackX) (Behrens *et al.* 2007) to reconstruct fibres passing through the region of interest (ROI) resulted from the above GLM analysis if high degree of cross fibres existed (see Associations with white matter fibre organization section). Tractography was performed in native diffusion space by transforming the ROI as seed masks from standard space into diffusion space using the inverse of the non-linear registration calculated in the TBSS pipeline. We used standard parameters (5000 samples/voxel, curvature threshold 0.2, step length 0.5 mm, samples terminated after 2000 steps or when they reached the surface as defined by a 40% probabilistic whole-brain white-matter mask). Connectivity maps of each individual were thresholded at 1% of total samples, mapped to standard space using non-linear registration and concatenated into a single 4D file.

## Results

### Categorizing experience

#### Binocular rivalry

Two metrics were calculated using data from the bistable perception session. The first was the mean duration (in seconds) of each period where one stimulus continuously dominated experience (dominance duration). Mean dominance duration shows robust and stable individual differences (Pettigrew and Miller 1998), which have previously been shown to be associated with connectivity between regions of parietal cortex (Baker *et al.* 2015), as well as the concentration of inhibitory neurotransmitters (gamma-aminobutyric acid, GABA) in visual regions of the brain (van Loon *et al.* 2013). Dominance durations are also affected by various personality types (Antinori *et al.* 2017a,b) and clinical conditions including autism (Robertson *et al.* 2013), bipolar disorder (Pettigrew and Miller 1998; Miller *et al.* 2003) and schizophrenia (Xiao *et al.* 2018; Ye *et al.* 2019). The second metric was the time when neither percept dominated experience, and so corresponds to the amount of time that participants reported seeing both percepts (mixed). Mixed percepts occur at transitions between states of full dominance, and involve a network of frontal and parietal brain areas, particularly in the right hemisphere (Knapen *et al.* 2011). Measures of these metrics were then transformed into *z*-scores, with outliers (>2.5, and based on visualization of boxplot generated in SPSS 25) being replaced with mean values (number of outliers: ‘dominance duration’ = 23, ‘mixed’ = 11). We found no correlations on the scores of these two metrics (*r* = 0.02, *P* < 0.9).

#### Experience sampling

In our analysis, we used the decomposition reported by Sormaz *et al.* (2018, see original paper for complete details). In brief, PCA was applied to MDES data at the trial level as standardized in our other works (e.g. see Smallwood *et al.* 2016; Konishi *et al.* 2017). This produced four components: (i) ‘Detail’, reflecting patterns of detailed visual task-related experience, (ii) ‘Off-task thought’, dissociating on-task thoughts from episodic self-relevant thoughts, (iii) ‘Modality’, distinguishing thoughts related to images or words and (iv) ‘Emotion’ describing the affective tone of experiences. These components are presented in the form of word clouds in the right hand panel of Fig. 1.

### Associations with white matter fibre organization

**Figure 2.**
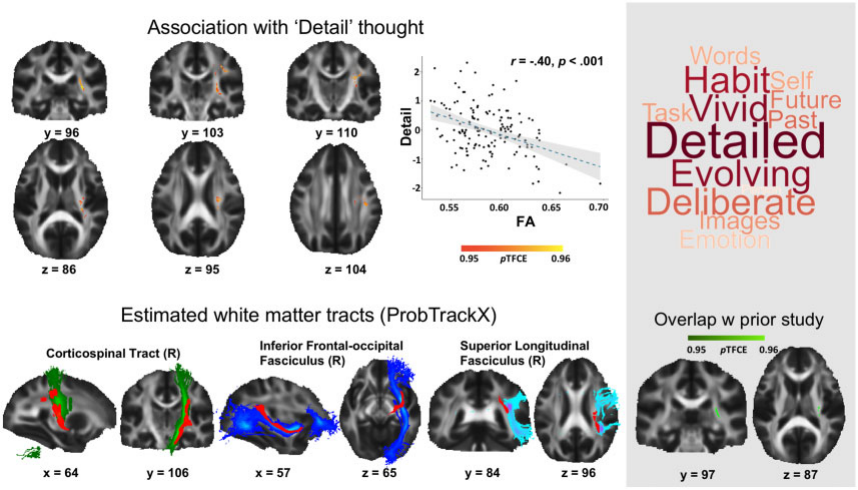
White matter tracts associated with less detailed external experience. Regions shown in red represent areas where FA (range 0–1) was higher (suggests greater structural connectivity) for individuals whose thoughts lacked vivid details. In the scatterplot (top left), each point represents an individual. The grey panel on the right shows the pattern of responses that reflect high levels of external detail (top right) and the overlap between the result in the current study with our prior study (bottom right). ProbTrackX analysis for estimating the overlap tracks, in red, and the three most probable white matter tracts, are presented in the bottom left section. All brain maps were overlaid on the group’s mean FA map and corrected for FWE using TFCE thresholding method (*P* < 0.05 FWE-corrected)

Our first analysis examined associations between white matter connectivity and patterns of ongoing thought identified using MDES data. We conducted a multiple regression in which individual participant's skeleton wide FA map was the dependent variable. Individual’s scores for each of the experiential dimensions identified through PCA were explanatory variables. Age and gender were included as nuisance covariates. Significant negative associations between FA and detailed thoughts were identified, and regions showing this relationship are presented in red in Fig. 2.

Next, we examined the relationship between the current result and those from our prior study (Karapanagiotidis *et al.* 2017). In Karapanagiotidis *et al.* (which used a different set of participants), we identified a set of right-lateralized tracts with greater FA for individuals reporting more mental time travel. Comparison of the two FWE-corrected maps indicated an area of overlap (see bottom right panel in Fig. 2). Karapanagiotidis *et al.* found higher FA linked to experiences characterized by self-generated thoughts about the past and the future, while the current results highlighted lower FA was linked to more detailed assessments of the here and now. Together, these results provide converging evidence that right-lateralized white matter tracts are important for differences in internal versus external focus of attention. As this region of overlap has a high degree of crossing fibres, we used ProbTrackX to estimate the white matter bundles to which this was most likely to be related (see Materials and Methods section, bottom left panel of Fig. 2). It can be seen that the results of this process highlighted multiple large fibre bundles including the inferior occipital-frontal (IFOF) and the cortico-spinal tract (CST), and the superior longitudinal fasciculus (SLF).

### Associations between different features of conscious experience

Having documented associations between white matter structures and ongoing thoughts, we next examined (i) whether patterns of ongoing experience identified by MDES are related to the nature of experience as determined via binocular rivalry, and, if so, (ii) whether these relationships are linked with the associated white matter architectural differences in brain structure. Table 1 shows the zero-order relationships across this set of variables.

**Table 1.** Simple correlations between *z*-scored measures of ongoing experience (represented as the rows) and *z*-scored metrics of bistable perception (represented in the columns)

		‘Dominant’	‘Mixed’
Detail	*r*	−0.12	0.13
	*P*	0.13	0.13
Off-task	*r*	−0.07	0.05
	*P*	0.42	0.57
Modality	*r*	−0.07	0.01
	*P*	0.41	0.94
Emotion	*r*	−0.07	0.14
	*P*	0.42	0.09

*r* = Pearson correlation; *P *=* P*-value.

**Figure 3.**
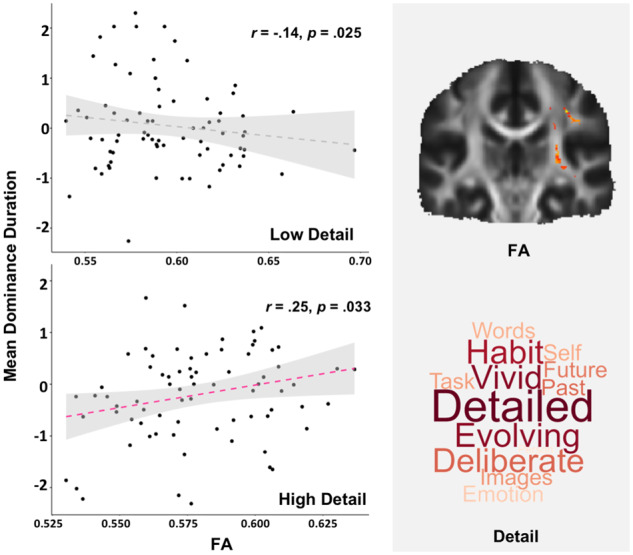
Association between individual variations in external detail in ongoing experience derived through experience sampling, the associated white matter architecture and visual experience determined via binocular rivalry. Scatterplots show that the longest periods of binocular rivalry dominance were experienced by participants with a combination of higher FA and reports of high external details in ongoing cognition (bottom left). For the purpose of display, the sample was splitted at the median value on their reported levels of 'Detail'. The grey panel on the right shows the areas where FA, associated with 'Detail' thoughts, was extracted (top right) and the pattern of responses that reflect high levels of external detail (bottom right)

To formally understand the relationship between different patterns of thought, their observed associations with white matter architecture, and the estimates of experience provided by binocular rivalry, we conducted a multivariate analysis of co-variance. In this analysis, mean dominance duration and the proportion of mixed percepts were the dependent variables. The explanatory variables were individual scores on each PCA dimension, as well as the DTI correlate of detailed experience (i.e. the mean FA for the white matter region that is correlated with 'Detail' experience). Age and gender were included as nuisance covariates. We modelled the main effect of each explanatory variable, as well as the interaction between ‘Detail’ and its white matter correlate. We found a significant interaction between ‘Detail’ and its association with white matter connectivity [*F*(2, 140) = 4.8, *P* = 0.011, partial eta squared = 0.06], reflecting differences in mean dominance duration [*F*(1, 149) = 7.43, *P* = 0.007, partial eta squared = 0.05]. To visualize this association, we plotted the relationship between FA separately for individuals with high and low 'Detail' experience (using median split). It can be seen that the shortest dominance durations were observed among individuals with high levels of 'Detail' and the lowest FA (see left hand panel of Fig. 3). Notably we did not find any association between off-task thought and binocular rivalry, nor with white matter architecture suggesting a relatively specific relationship with highly detailed externally focused experience.

## Discussion

Our study set out to better understand the neural basis of different types of shift in the quality of conscious experience by leveraging on methods of experience sampling, binocular rivalry and structural brain imaging. We found a correlation between individual differences in estimates of the integrity of cortical white matter in the right hemisphere and the level of detail with which external events were experienced. Notably, the pattern of right-lateralized white matter tracts that had greater integrity for less detailed experiences in the current analyses overlapped with our prior analysis using a different sample. Our prior study highlighted greater FA for individuals with a greater focus *away* from the moment to other times and places (Karapanagiotidis *et al.* 2017). Given that external focus is reduced during periods of self-generated imaginative thought (Kam *et al.* 2011; Kam and Handy 2013), these two results help establish the importance of a right-lateralized network of white matter tracts in determining aspects of cognition as assessed by experience sampling.

We found an interaction between the measure of detailed experience and its associated white matter correlate with patterns of dominance reported during bistable perception. In particular, individuals who reported higher levels of external details during sustained attention and had the lowest estimates of white matter integrity in these right-lateralized regions, also reported shorter periods when one percept dominated. It is usually assumed that, during binocular rivalry, top-down processes stabilize one potential interpretation of visual input, and so shorter time during rivalry is related to bottom-up influences on perception. Based on our data, our participants’ reports of detailed experience during sustained attention might emerge because of a conscious emphasis on bottom-up influences derived from sensory input that is, in turn, partly constrained by the white matter architecture of the cortex.

In this context, it is interesting to note that our prior studies found that neural signals within the DMN (traditionally assumed to be linked to internal states) encode patterns of detailed thoughts in a working memory task (Sormaz *et al.* 2018), while Turnbull *et al.* (2019b) found—in the same cohort as we report here—that, at rest, greater functional connectivity between the DMN and with visual cortex predicted more detailed experiences in the laboratory. Our recent work on the macrostructural organization of the cortex suggests that the DMN is functionally and spatially isolated from sensory and motor systems (Margulies *et al.* 2016). Prior studies suggest that the DMN can lead to a greater focus on external information with greater detail (Sormaz *et al.* 2018; Turnbull *et al.* 2019a,b), or, during efficient task performance (e.g. Esterman *et al.* 2013; Kucyi *et al.* 2016; Vatansever *et al.* 2017a). Perhaps, therefore, these examples (Esterman *et al.* 2013; Kucyi *et al.* 2016; Sormaz *et al.* 2018; Vatansever *et al.* 2018; Turnbull *et al.* 2019a,b) reflect situations when there is a particularly strong representation of bottom-up sensory signals within the DMN. Intriguingly, recent retinotopic mapping studies have identified that regions of the DMN can show patterns of selective deactivation as a function of the location of a visual stimulus (Szinte *et al.* 2019).

### Limitations and future directions

Although our study suggests a relationship between detailed processing of external information, the white matter architecture of the cortex and patterns of dominance during rivalry, there are a number of important limitations that should be borne in mind when considering these results. First, based on our data, it seems possible that fluctuations in the degree of task-relevant attention during binocular rivalry will impact upon the nature of how external information can dominate at a given moment in time. Our data cannot address this issue directly because we did not measure experience during the binocular rivalry session. It will be important in the future to measure the focus of individuals’ experience while they are exposed to rivalrous stimuli to address this possibility. Second, alternative measures of tractography are able to detect non-Gaussian features of FA (Cohen-Adad *et al.* 2008) and it may be worthwhile using these metrics in future studies examining associations with cognition and the white matter structure of the cortex. Third, it is possible that the measure of rivalry, which depends on the participants’ ability to recognize the switches in their conscious experience, may under-represent the actual number of shifts, particularly for participants who lack meta-awareness of their ongoing thought patterns (Schooler 2002). In future studies, this limitation could be addressed by intermittently probing individuals to determine which percept they were currently consciously attending to.

We close by considering the possibility that the fibre bundles identified through probabilistic tractography in our study may offer a possible window into how the DMN can contribute to modes of operation that have both internal and external features. An emerging puzzle in cognitive neuroscience is the role that the DMN plays in cognition. Initial views of this system suggested that it was linked primarily to internal states of ongoing experience that were broadly unrelated to external task performance (e.g. Fox *et al.* 2005). However, evidence implicating this system in external tasks (Esterman *et al.* 2013; Smallwood *et al.* 2013; Konishi *et al.* 2015; Vatansever *et al.* 2015, 2017b; Murphy *et al.* 2018, 2019) coupled with our prior demonstrations of a role of the DMN in patterns of detailed thought (Smallwood *et al.* 2016; Sormaz *et al.* 2018; Turnbull *et al.* 2019a,b) challenge the views of this large-scale system as important for purely internal thoughts. Our study identified a white matter region linked to patterns of detailed external thought that was at the overlap of three major white matter fibre bundles. The CST, which originates in regions of sensory and motor cortex with most axons crossing at the anatomical midline between brainstem and spinal cord, is the principal motor pathway for voluntary behaviour and is important for the modulation of sensory information (Kolb and Whishaw 2009; Welniarz *et al.* 2017). The SLF is a major white matter pathway that connects the frontal, parietal, temporal and occipital lobes (Kawamura and Naito 1984; Petrides and Pandya 1984; Makris *et al.* 2004; Bernal and Altman 2010); although it has often been associated with playing a key role in language function, together with the arcuate fasciculus, its precise functional role remains disputed (Wang *et al.* 2016). For example, it is now recognized to make a contribution to broader functions of working memory and cognitive control (e.g. Vestergaard *et al.* 2011; Chaddock-Heyman *et al.* 2013). The IFOF is important for connecting the superior frontal and parietal cortices (Hau *et al.* 2016). It is impossible to determine precisely which of these tracts has the most important link with experience because of limitations of the ability of DTI to distinguish crossing fibres (Jbabdi *et al.* 2010); however, emerging evidence suggests that these three tracts may be reasonable candidates for future studies to explore. For example, recent works have suggested that the microstructural architecture of the SLF is predictive of patterns of unpleasant brooding in depression and functional connectivity of a precuneal network within the broader DMN (Pisner *et al.* 2019), as well as the perception and experience of emotions (Ho *et al.* 2016). Similarly, Bonnelle *et al.* (2012) found that traumatic brain injury to a white matter path identified by probabilistic analysis led to less efficient regulation of neural activity within the DMN by the saliency network. Notably, we recently demonstrated that the saliency network plays a critical role in the adaptive allocation of conscious attention to both internal and external foci, in part through its relationship to both the DMN and to systems important for external attention, namely the dorsal attention network (Turnbull *et al.* 2019a). In future, it would be useful to explore how the structural architecture of the brain constrains the functional activity in the cortex and, in particular, the DMN, across situations varying in their reliance on internal and external modes of cognition.

## Conclusions

Prior studies have implicated the DMN in task-relevant material, in particular, in experiences with an emphasis on detailed representations of task-relevant information. The current study combined experience sampling, measures of white matter architecture and indices of binocular rivalry. We found that a detailed focus on task-relevant information during sustained attention was linked to the integrity of white matter pathways in the right hemisphere. This association was linked to shorter periods of dominance during binocular rivalry. Together these results suggest that detailed representations of external task-relevant information may be associated with conscious emphasis on bottom-up influences derived from sensory input, that is possibly constrained by the white matter architecture of the right hemisphere. Our study highlights the possibility that although the DMN is traditionally assumed to be linked to internal states, it may also be associated with task-relevant information under situations when there are particularly strong representation of bottom-up sensory signals in transmodal cortex.

## Supplementary data

Supplementary data is available at *NCONSC Journal* online.

## Supplementary Material

niaa020_Supplementary_Data
